# The Influence of Sub-Unit Composition and Expression System on the Functional Antibody Response in the Development of a VAR2CSA Based Plasmodium falciparum Placental Malaria Vaccine

**DOI:** 10.1371/journal.pone.0135406

**Published:** 2015-09-01

**Authors:** Morten A. Nielsen, Mafalda Resende, Willem A. de Jongh, Sisse B. Ditlev, Benjamin Mordmüller, Sophie Houard, Nicaise Tuikue Ndam, Mette Ø. Agerbæk, Mette Hamborg, Achille Massougbodji, Saddou Issifou, Anette Strøbæk, Lars Poulsen, Odile Leroy, Peter G. Kremsner, Jean-Philippe Chippaux, Adrian J. F. Luty, Philippe Deloron, Thor G. Theander, Charlotte Dyring, Ali Salanti

**Affiliations:** 1 Centre for Medical Parasitology at Department of Immunology and Microbiology, Faculty of Health and Medical Science, University of Copenhagen, and Department of Infectious Diseases, Copenhagen University Hospital (Rigshospitalet), Copenhagen, Denmark; 2 ExpreS^2^ion Biotechnologies, SCION-DTU Science Park, Hørsholm, Denmark; 3 Eberhard Karls Universität Tübingen, Institut für Tropenmedizin, Tübingen, Germany, and Centre de Recherches Médicales de Lambaréné, Lambaréné, Gabon; 4 European Vaccine Initiative, Universitäts Klinikum Heidelberg, Heidelberg, Germany; 5 Institut de Recherche pour le Développement, UMR216 Mère et enfant face aux infections tropicales, Paris, France; 6 PRES Sorbonne Paris Cité, Université Paris Descartes, Paris, France; 7 Faculté des Sciences de la Santé de l’Université d’Abomey-Calavi, Centre d’étude et de recherche sur le paludisme associé à la grossesse et à l’enfance, Cotonou, Bénin; London School of Hygiene and Tropical Medicine, UNITED KINGDOM

## Abstract

The disease caused by *Plasmodium falciparum* (Pf) involves different clinical manifestations that, cumulatively, kill hundreds of thousands every year. Placental malaria (PM) is one such manifestation in which Pf infected erythrocytes (IE) bind to chondroitin sulphate A (CSA) through expression of VAR2CSA, a parasite-derived antigen. Protection against PM is mediated by antibodies that inhibit binding of IE in the placental intervillous space. VAR2CSA is a large antigen incompatible with large scale recombinant protein expression. Vaccines based on sub-units encompassing the functionally constrained receptor-binding domains may, theoretically, circumvent polymorphisms, reduce the risk of escape-mutants and induce cross-reactive antibodies. However, the sub-unit composition and small differences in the borders, may lead to exposure of novel immuno-dominant antibody epitopes that lead to non-functional antibodies, and furthermore influence the folding, stability and yield of expression. Candidate antigens from the pre-clinical development expressed in High-Five insect cells using the baculovirus expression vector system were transitioned into the *Drosophila* Schneider-2 cell (S2) expression-system compliant with clinical development. The functional capacity of antibodies against antigens expressed in High-Five cells or in S2 cells was equivalent. This enabled an extensive down-selection of S2 insect cell-expressed antigens primarily encompassing the minimal CSA-binding region of VAR2CSA. In general, we found differential potency of inhibitory antibodies against antigens with the same borders but of different *var2csa* sequences. Likewise, we found that subtle size differences in antigens of the same sequence gave varying levels of inhibitory antibodies. The study shows that induction of a functional response against recombinant subunits of the VAR2CSA antigen is unpredictable, demonstrating the need for large-scale screening in order to identify antigens that induce a broadly strain-transcending antibody response.

## Introduction

Malaria is caused by the blood stages of *Plasmodium spp*. It is a systemic disease inducing an intense inflammatory response that manifests as fever, nausea, headache, and myalgia but may also lead to organ failure and life-threatening metabolic disturbances. Although preventable and treatable the most severe manifestations of malaria due to *P*. *falciparu*m cause more than half a million deaths per year, predominantly in sub-Saharan African populations [1].

The majority of malaria deaths is caused by Plasmodium falciparum that, in contrast to other *Plasmodium* species, sequester in the deep vasculature of various tissues including the brain, lung, bone-marrow and the placenta (reviewed in [2]). Malaria-naïve individuals are at high risk to develop potentially fatal symptoms, whilst in highly endemic regions the groups most at risk of developing severe malaria syndromes are young children and pregnant women. Importantly, the scale-up in current control measures, including bed-nets and intermittent preventive treatment appear to have contributed to a reduction in transmission [1]. As current control measures are hampered by high costs, development of drug resistance and a need for a sustained effort over many years, vaccination could be a cost-effective complement to current control measures [3]. Many malaria vaccine candidates target the pre-erythrocytic stages of the infection, among these RTS,S is the leading vaccine, which is entering a licensing process [4]. Furthermore, efforts are being made to develop transmission blocking vaccines based on parasite antigens expressed in the sexual stages, both in humans and mosquitoes [5]. However, these vaccines may need to be combined with other candidates targeting the blood-stage, aiming not at eradicating infection but deaths and morbidity due to malaria [6].

Theoretically, the antigens that mediate adhesion of IE to the blood microvasculature, namely *P*. *falciparum* erythrocyte membrane protein 1 (PfEMP1) family members, are very promising vaccine candidates. However, the *var* genes that encode PfEMP1 are polymorphic and naturally-acquired protection relies on the infection-induced acquisition of a broad repertoire of anti-PfEMP1 antibodies during early childhood [7,8]. Therefore the increased risk of infections during pregnancy, especially in primi-gravidae, was for a long time enigmatic. A major breakthrough was the discovery that the conserved PfEMP1 antigen VAR2CSA enables IE to bind to chondroitin sulphate A (CSA) on syncytiotrophoblasts and thereby to accumulate in the placenta [9]. Apparently, the tropism for placental CSA of VAR2CSA restricts expression of this molecule to infections of pregnant women, as children and men in malaria endemic areas have very low levels of antibodies to VAR2CSA [10–12]. The lack of protective antibodies and the tropism for CSA of VAR2CSA hence explains the vulnerability of pregnant women [13]. As protection against PM is associated with antibodies that inhibit the binding of infected erythrocytes to CSA [14,15], the primary goal is the development of an adhesion-blocking vaccine, although opsonizing antibodies may also play a role in protection [16]. The *var2csa* gene is among the most conserved *var* genes and pre-clinical evaluation of the cross-reactivity of antibodies against different VAR2CSA antigens has shown promising results [11–16]. Furthermore, the identification of the minimal binding region of VAR2CSA increased the hypothetical possibility of identifying an antigen less prone to immune escape by mutation, to include in the clinical development of a placental malaria vaccine [17].

The development of a placental malaria vaccine aligns with the WHO Malaria Vaccine Technology Roadmap, with the strategic goal to have by year 2030 a licensed malaria vaccine with at least 75% efficacy in an at-risk population [18], which in the context of placental malaria is the neonate. Our early clinical testing will comprise a multi-center Phase 1 clinical trial both in healthy adult European and African volunteers. The Phase 1a trial will be performed in Europe (Tübingen, Germany) in healthy, malaria-naïve, adult volunteers. The objective will be selection of a dose and formulation that optimizes the balance between immunogenicity, tolerability and safety. The Phase 1b trail will be performed in Africa (Calavi, Bénin). Here the study population will consist of healthy, adult, nulligravid female volunteers with life-long exposure to malaria. The primary objective of the trial will be the assessment of the safety and tolerability of the vaccine. Secondary objectives of both arms of the trial will be the assessment of the immunogenicity of the VAR2CSA vaccine candidate. The humoral immune response will be assessed by measuring (i) the level of vaccine antigen-specific total IgG by ELISA, (ii) the ability of antibodies to recognize the native VAR2CSA protein expressed on the surface of IE by flow-cytometry and the ability of induced IgG to inhibit the binding of IE to CSA *in vitro*.

This paper describes the selection of the antigen to include in the clinical development of a placental malaria vaccine. The selection was based on assessments of the *in vitro* functional capacity of immunization-induced antibodies using the same assay that will be used in the clinical development phase. The influence of sub-unit composition on the functional antibody response is tested after transition of antigens from the baculovirus expression system in High-Five cells to the “current Good Manufacturing Practice” (cGMP)-compliant *Drosophila* Schneider-2 (S2) expression system.

## Results

### Comparison of the ability of antibodies against *Drosophila* S2 and *Trichoplusia ni* High-Five derived antigens to inhibit CSA binding

**Fig 1:**
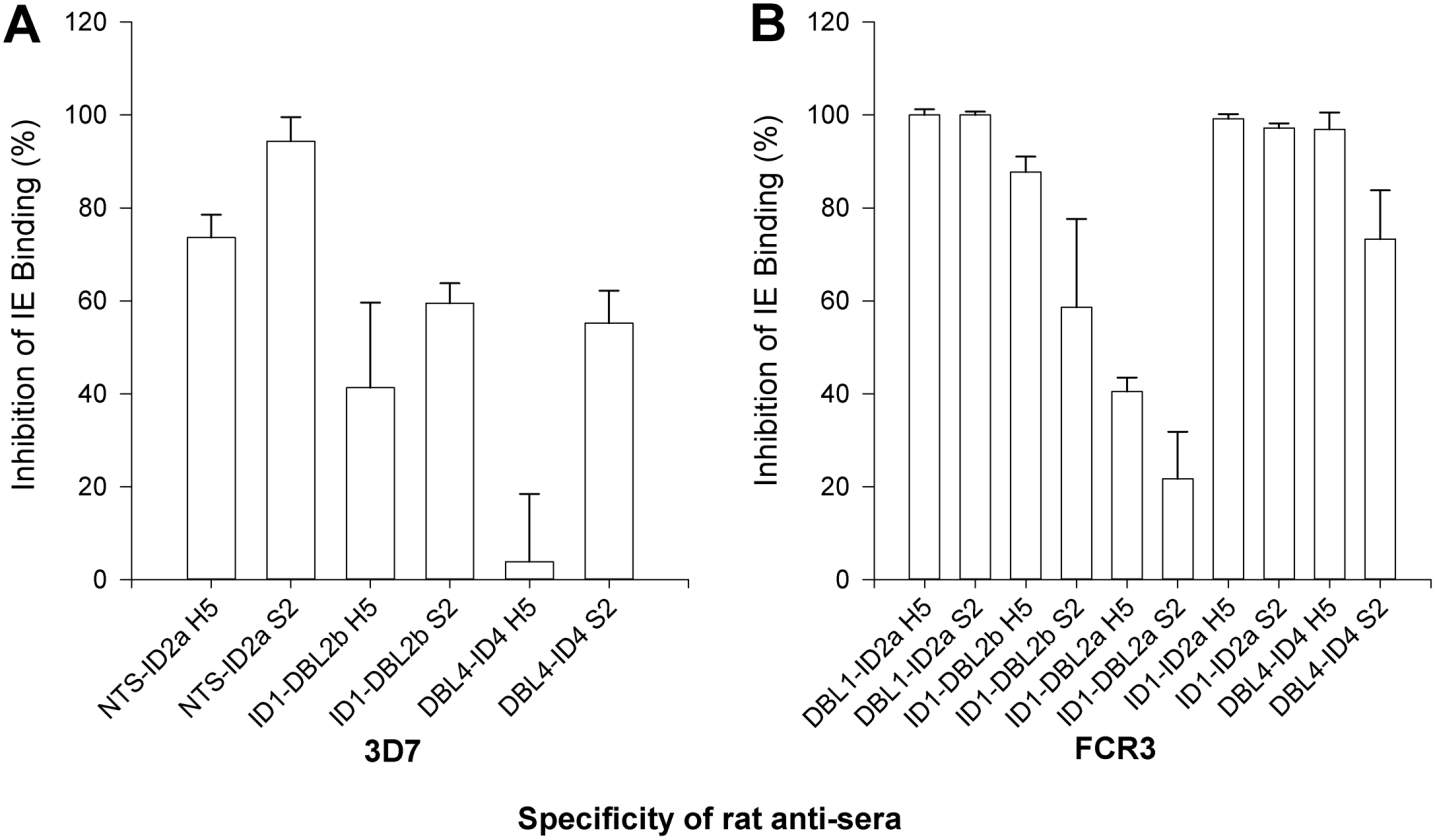
Comparison of the induction of binding inhibitory antibodies against antigens expressed in High-Five or S2 cells. Bars indicate the mean binding inhibition of 3D7 (A) or FCR3 (B) *Plasmodium falciparum* infected erythrocytes (IE). The values are the percentages of released IEs in wells with test serum compared to wells with control serum from non-immunized animals. Serum was used in a one to ten dilution. Error bars indicate the coefficients of variation of the mean of triplicate measurements (100*(SD/(mean of control binding)).

One of the challenges of recombinant expression of PfEMP1 molecules is the structural complexity of the antigens, thus several expression systems have been explored previously [19]. In previous pre-clinical development efforts we have worked with High-Five cells using the baculovirus expression vector system [20]. Insect cells enable expression of large proteins with multiple disulfide bonds without the need for refolding. As the baculovirus system, from a regulatory point of view, is not ideal for cGMP production we transitioned antigen expression to cGMP qualified S2 cells using a plasmid transfection system proprietary to ExpreS^2^ion Biotechnologies, thus avoiding virus infection. As this was a previously unexplored expression system for malaria antigens, we wanted to evaluate this transition in terms of the functional antibody response. Originally the plan was to make head-to-head comparisons of antibodies raised by immunizations with all antigens expressed in S2 or High-Five cells. However, a direct comparison of antigens was only possible for eight antigen pairs (Table 1 and Fig 1), as not all antigens could be expressed in both systems (Data not shown). The *in vitro* efficacy of antibody-specific inhibition of homologous IE binding to CSA was used to evaluate the potency of the vaccine and showed equivalence in the immunogenicity of antigens expressed in High-Five cells or in S2 cells (Fig 1)(Wilcoxon: P = 0.9).

**Table 1:** Expressed recombinant proteins and adjuvants used in immunizations.

Genotype	Domain borders	Amino-acid interval	Expression System	Adjuvant
**3D7**	NTS-DBL2a	N8-E865	S2 cells	Alum
	NTS-DBL2b	N8-Y953	S2 cells	FCA
	NTS-DBL2a	N8-E865	S2 cells	FCA
	NTS-ID2a	N8-D1016	High-Five	FCA
	NTS-ID2a	N8-D1016	S2 cells	FCA
	DBL1-ID2b	H57-S1206	High-Five	FCA
	ID1-DBL2b	N385-Y953	S2 cells	FCA
	ID1-DBL2b	L400-Y953	High-Five	FCA
	ID1-ID2a	N385-D1016	S2 cells	FCA
	ID1-ID2b	L400-S1206	High-Five	FCA
	ID1-DBL4	L400-D1983	High-Five	FCA
	DBL2-DBL4	L537-D1983	High-Five	FCA
	DBL4-ID4	E1565-R1984	S2 cells	FCA
	DBL4-ID4	E1565-D1983	High-Five	FCA
**FCR3**	NTS-DBL2a	N9-A869	S2 cells	FCA
	DBL1-DBL2b	H58-Y962	High-Five	FCA
	NTS-DBL2b	M1-K967	E.coli	Alum
	DBL1-ID2b	H58-K1214	High-Five	FCA
	NTS-ID2a	M1-D1059	E.Coli	Alum
	NTS-ID2a^(N-Glyc-Mut)^	N9-D1025	S2 cells	FCA
	DBL1-DBL2a	H58-A869	S2 cells	FCA
	DBL1-ID2a	H58-D1025	S2 cells	FCA
	DBL1-ID2a	H58-D1025	High-Five	FCA
	ID1-DBL2a	N386-A869	S2 cells	FCA
	ID1-DBL2a	N386-A869	High-Five	FCA
	ID1-DBL2b	N386-Y962	S2 cells	FCA
	ID1-DBL2b	N386-Y962	High-Five	FCA
	ID1-DBL2b^(N-Glyc-Mut)^	N386-Y962	High-Five	FCA
	ID1-ID2a	N386-D1025	S2 cells	Alum
	ID1-ID2a	N386-D1025	E.coli	Alum
	ID1-ID2a	N386-D1025	High-Five	FCA
	ID1-ID2a	N386-D1025	S2 cells	FCA
	ID1-ID2a^(N-Glyc-Mut)^	N386-D1025	S2 cells	FCA
	DBL4-ID4	K1583-D1989	S2 cells	FCA
	DBL4-ID4	K1583-D1989	High-Five	FCA

### The cross-reactivity of antibodies against S2 derived recombinant antigens

**Fig 2:**
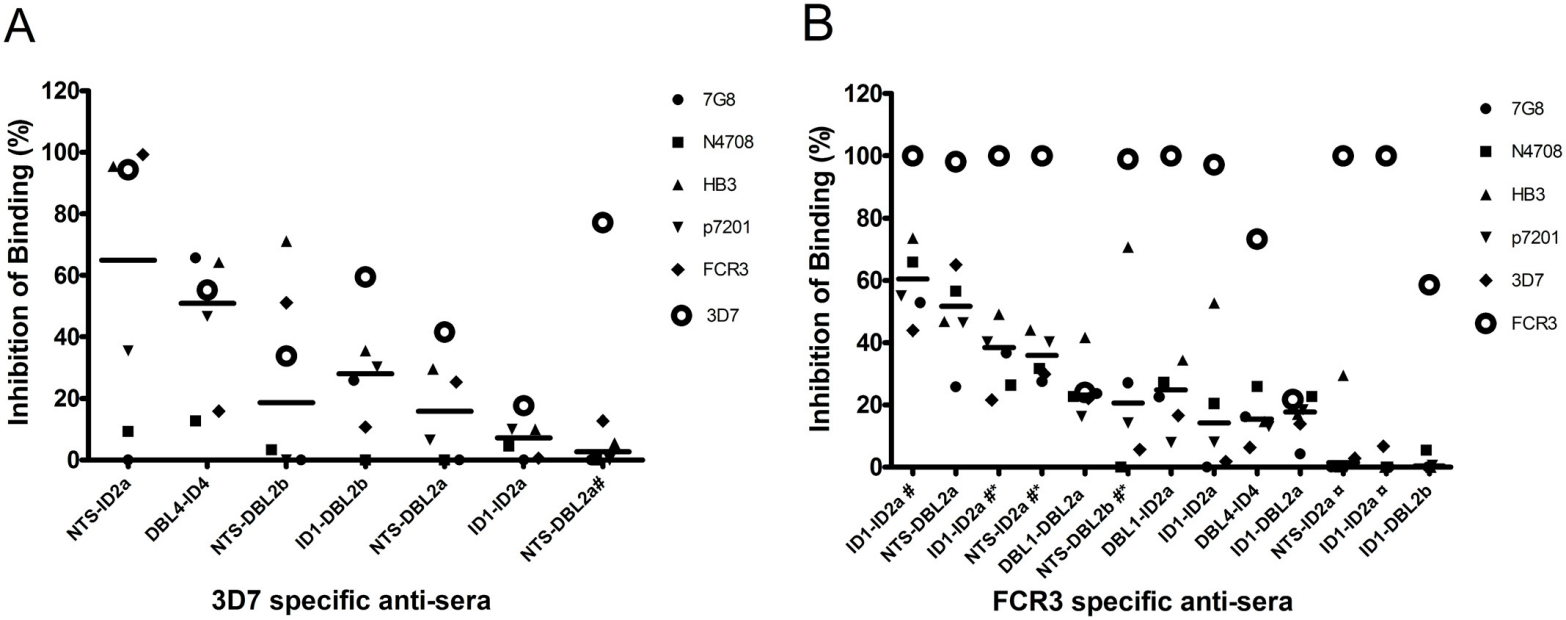
Cross reactivity of 3D7 and FCR3 antigens expressed in S2 cells and E. coli. The symbols indicate the mean binding inhibition by serum pool from rats immunized with S2 antigens in Freund’s adjuvant unless otherwise indicated. Antigens were derived from either 3D7 (A) or FCR3 (B) *var2csa* genes (see Table 1). The binding inhibition of sera was tested against *Plasmodium falciparum* infected erythrocytes 3D7 (A) or FCR3 (B) and a panel of heterologous parasites (7G8, N4708, HB3, P7201). The mean binding inhibition values are indicated as the percentages of binding inhibited IEs in wells with test serum compared to wells with control serum from non-immunized animals. Serum was used in a one to ten dilution. Horizontal lines indicate the mean percentage of binding inhibition of heterologous isolates. # indicates immunizations using Alhydrogel. * indicates antigens expressed in *E*.*coli* SHuffle. ¤ indicates glycosylation mutants.

The *var2csa* gene has limited sequence variation as compared to other *var* genes and in areas of high transmission, a substantial priming of VAR2CSA-specific memory B-cells probably occurs during the first pregnancy, since an increase in the mean birth-weight of babies born in the following pregnancies is associated with an increased capacity of naturally acquired antibodies to inhibit the binding of IEs to CSA [13,14]. To address the cross-reactivity of antibodies against vaccine candidates differing by sequence (FCR3 and 3D7), domain number and domain borders, the average inhibition of a panel of five parasite isolates (see Materials and Methods) was assessed (Fig 2A and 2B). The antibodies were derived from immunizations of rats in groups of three with S2-produced antigens derived from either the 3D7 or FCR3 genotypes. As the ability to generate cross-reactive antibodies and the yield of production in S2 was unknown at the time of protein expression a limited number of antigens were also expressed in *E*. *coli* shuffle cells as a contingency plan (Fig 2B).

We found that both the homologous and the cross-inhibitory reactivity of antibodies appeared sensitive to subtle changes in domain composition. Furthermore, antigens encompassing the same VAR2CSA subunits, but of different parasite genotype origin, induced different levels of cross-reactive antibodies. For instance, the most cross-reactive 3D7-specific antibodies were raised against the NTS-ID2a antigen, but this was not the case for FCR3 sequence-derived NTS-ID2a, while the opposite appeared to be the case for the FCR3 and 3D7 sequence derived ID1-ID2a antigen (Fig 2A and 2B). In addition, it appeared that mutations to reduce glycosylation had a negative effect on cross-reactivity of the induced antibody response (Fig 2B: FCR3-S2-NTS-ID2a & FCR3-S2-ID1-ID2a), although antigens expressed in *E*. *coli*, non-glycosylated *per se*, appeared to induce a fully functional response (Fig 2B: FCR3-E.Coli-NTS-ID2a & FCR3-E.coli-ID1-ID2a). Importantly, the general trend was that efficacious inhibition of the homologous isolates appeared to predict activity against the heterologous isolates, although exceptions were identified (3D7-NTS-DBL2 & FCR3-DBL1-DBL2b (Fig 2A and 2B).

### Selecting a high-expressing monoclonal S2 cell line to maximize expression yields

**Fig 3:**
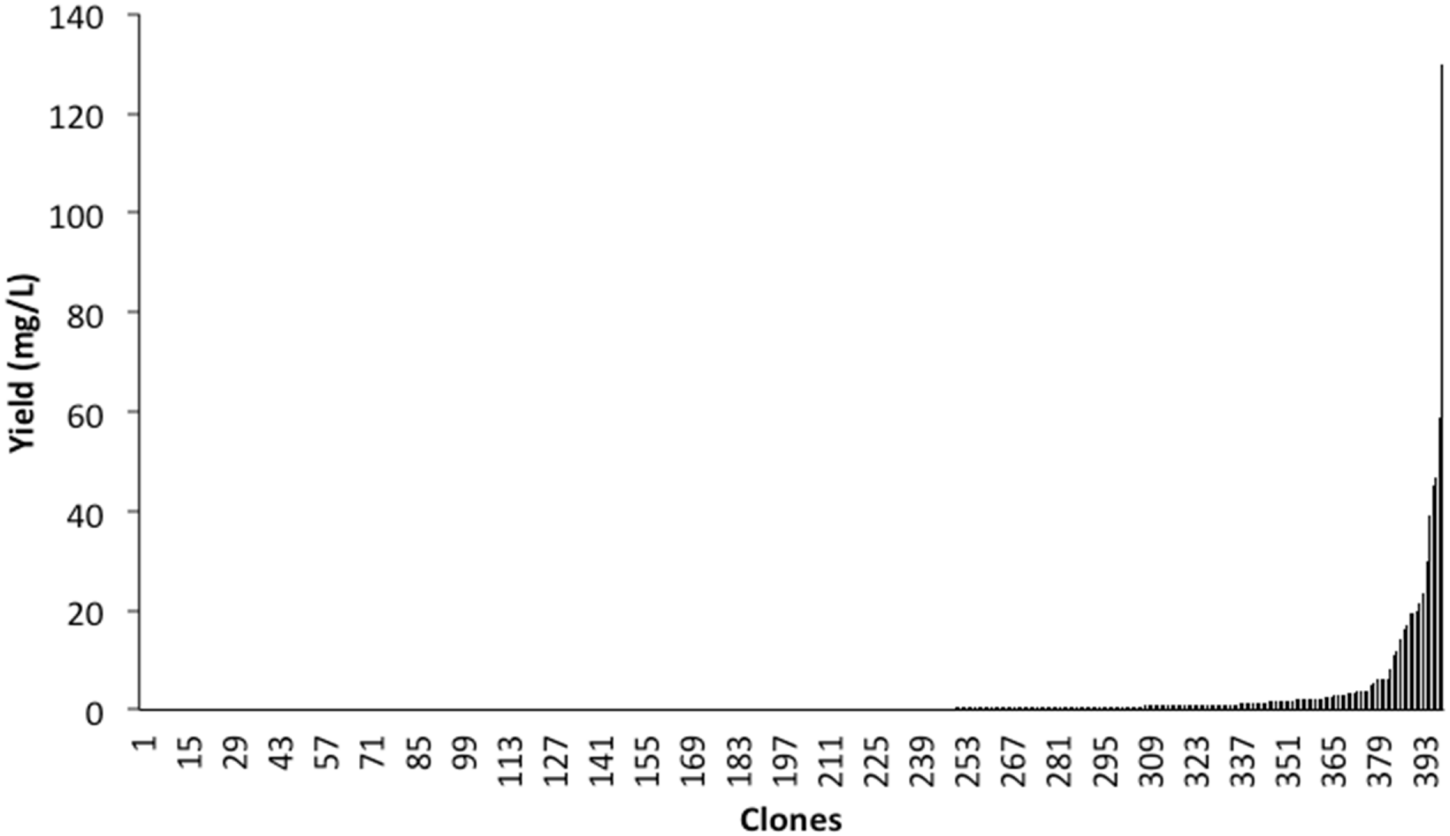
Expression levels of ID1-ID2a producing S2 monoclonal cell lines. Bars indicate the yield of protein in the culture supernatant of stable transfected S2 clones. The values were determined by testing titrations of culture supernatant compared to a standard protein solution in sandwich ELISA using one monoclonal ID1-ID2a specific antibody to capture and another monoclonal ID2-ID2a specific antibody as a the target of a tertiary layer of horse radish peroxidase conjugated antibodies.

The production cost of a vaccine is especially important for products targeting diseases in poor populations, where cost per dose is a factor of high relevance to vaccine success. As the ID1-ID2a-FCR3 antigen appeared superior in terms of induction of a functional homologous and heterologous binding-inhibitory antibody response, we selected this candidate for further development. We screened 393 monoclonal cell lines expressing the ID1-ID2a antigen without an His-tag to select the highest expressing clones. As expected, we observed a large variation with respect to expression levels in different monoclonal S2 cell lines in 12-well format, with a large proportion of the cell lines producing less than 1mg/L (Fig 3A). After selection and optimization, the monoclonal cell line selected for clinical material manufacture had an expression level in excess of 150mg/L (Fig 3) and was stable for at least 30 generations (data not shown).

## Discussion

A number of different VAR2CSA vaccine constructs were identified using different expression systems in the effort to develop a placental malaria vaccine [7–12]. Previously, we found that the level of CSA binding inhibitory antibodies varied independent of the levels of antibodies measured by ELISA [21,22]. The reason for the differential fine specificity of antibodies in different immunizations is unknown, but differential glycosylation patterns and/or differences in folding of the antigens from lot to lot, and in different expression systems, could be the cause of the variation. As we had not previously used the S2 expression system, it was necessary to perform an extensive screening of the antibody-mediated inhibition of binding to CSA of both homologous and heterologous parasite isolates.

In this study, antibodies against several vaccine candidates expressed in S2 cells E. coli, and primarily encompassing the minimal binding region of VAR2CSA, were tested for the ability to inhibit binding of IE to the placental receptor CSA. In general, we found that the domain composition and borders of sub-units of the most potent antigens with regard to induction of a potent of inhibitory antibody-response were not the same for the FCR3 and 3D7 *var2csa* sequences. This could be caused by the exposure of immuno-dominant epitopes targeted by non-inhibitory antibodies in one *var2csa* sequence variant compared to other sequence variants. As more than one variant may be needed in order for a vaccine to be fully protective, this could have implications for further clinical development. We found that for antigens of the same sequence, subtle differences in domain borders could have an impact on the induction of functional antibodies. Although in the current study we could not detect significant differences of vaccine potency originating from either of the two expression systems used, the inability to express all antigens with equal efficiency in both systems, underlines the importance of selecting an expression system compatible with clinical development early in the antigen selection process. The repertoire of protective antibodies, i.e. the number of VAR2CSA variants, that a protected individual has generated antibodies to, is currently unknown. However, the naturally acquired antibody response appears not to be generated uniformly against individual VAR2CSA domains [23–25], which could indicate the presence of immuno-dominant B-cell epitopes that may be non-protective. Priming the immune response with a vaccine that target the minimal binding region may reduce the acquisition of non-functional antibodies during subsequent natural infections. Based on the ability to generate cross-reactive antibodies the ID1-ID2a-FCR3 antigen appeared the best, closely followed by the NTS-DBL2-FCR3 antigen. However, the NTS-DBL2-FCR3 does not bind to CSA and contains one domain more than the ID1-ID2a antigen increasing the risk of containing non-functional B-cell epitopes [17]. Based on this, the ID1-ID2a antigen was chosen for clinical development. Although the primary endpoint in the early phases of clinical development is safety, the secondary and exploratory endpoints for our phase 1 trial include testing the induction of functional binding-inhibitory antibodies, the cross reactivity, as well as the ability to generate specific immune memory, providing evidence for the possibility of vaccination of adolescent girls. This will enable decisions for further clinical development that will also rely on demographic and anthropological studies performed in young nulli- or older multi-gravid women, in households and amongst opinion leaders to identify the target population.

## Materials and Methods

### Parasite culture

Parasites were maintained using 5% hematocrit of human blood group 0+ blood in RPMI 1640 (Sigma) supplemented 0.125 μg/ml Albumax II (Invitrogen), and 2% normal human serum as described [20]. Laboratory isolates FCR3, NF54, HB3 and 7G8 and placental tissue derived parasites p7201 and N4708 were selected for binding to CSA by panning on BeWo cells as described [26]. Parasite isolates were regularly genotyped using nested GLURP and MSP-2 primers in a single PCR step, as described [27], and tested negative using a mycoplasma contamination kit (Lonza).

### Protein production

FCR3 and 3D7 *var2csa* were made as expression-optimized genes at GeneArt (LifeTechnologies). Domain and sub-domain borders of *var2csa* were determined by structural and DNA sequence analysis [28,29]. Gene fragments were cloned into the baculovirus vector pAcGP67-A (BD Biosciences), or pExpreS2-1 plasmid (Expres2ion) and E. coli pEt plasmid (Novagen) containing a V5 epitope upstream of a histidine tag in the C-terminal end and used to transfect Drosophila S2 (ExpreS^2^ion) or *E*. *coli* SHuffle (New England Bio labs) cells or to make a recombinant baculovirus using linearized BacPack DNA that was transfected into Sf9 insect cells to generate a recombinant baculovirus.

For the baculovirus expression system High-Five insect cells were grown in 600 ml of serum-free media (10486; GIBCO) and infected with 18 ml of the second amplification of the recombinant virus particles. After 2 days of induction, the cells were centrifuged (8000g, 4°C, 10 min), and the supernatant was filtered using two 10-kDa NMWC PES membranes (0.45 μm) (56-4112-04; GE Healthcare). The supernatant was then diafiltrated six times on an ÄKTA cross-flow (GE Healthcare) and loaded onto a 1-ml HisSelect column (H8286; Sigma-Aldrich) using an ÄKTA-express purification system (GE-Healthcare). The bound protein was eluted with buffer A + 200 mM imidazole (HisSelect) and loaded directly onto a size exclusion chromatography HiLoad Superdex 200PG (28-9893-35, GE Healthcare). Monomeric peak was concentrated using Vivaspin 20 columns (28-9323-60, GE Healthcare). Stable polyclonal S2 cell lines were established for each of the var2csa protein variants according to a similar protocol as reported in Wright et al. 2014 [30]. Production of S2 proteins was performed in 2L shake flasks inoculated to 8E6 cells /ml in 500ml serum-free Excell420 culture medium (SIGMA), shaken at 110rpm, controlled to 25°C, and harvested after 4 days of cultivation. S2 protein purification was performed similarly to the baculovirus expressed batches. For the E. coli Shuffle protein expression cells were harvested by centrifugation at 10,000 x g for 10 minutes. Cell pellet was resuspended in 20 mL lysis buffer (20mM NaPO4 pH7.2, 0.5M NaCl, 20mM imidazole, 1 Complete Mini EDTA-free protease inhibitor cocktail tablet (Roche, 11836170001)) and sonicated on ice at 70% power with 5 pulsations for 2 x 4 minutes. Cell lysate was centrifuged at 40,000 x g at 4°C for 30 minutes, and the supernatant was 0.2μm filtered followed by Ni Affinity chromatography and size exclusion similar to the insect cell produced proteins. The proteins produced by the baculovirus transfected insect cells as well as the S2 insect cells were designed to be secreted into the culture supernatant during expression. Such proteins have been processed through ER and Golgi and contain putative glycosylations and disulfide bonds. The recombinant proteins were purified from the culture supernatant with no further modifications of the structure (i.e. no refolding). The E. coli Shuffle cells are genetically engineered to produce recombinant proteins in the cytoplasm of the cell in a modified environment to promote disulfide bond formation. Recombinant protein was purified from the cytoplasm of the Shuffle cells with no further modifications (i.e no refolding).

All proteins were tested by SDS page to confirm purity (S1 Fig).

### SDS-PAGE

Fifteen microliters of each purified protein fraction were mixed with 3 μl of 6x Loading dye (300 mM Tris/HCl pH6,8; 12% SDS; 60% glycerol; 0,6% bromophenol blue) and heated at 95°C for 5 minutes followed by loading to a precast SDS-PAGE gel (NuPAGE 4–12% Bis-Tris, Life technologies) for electrophoresis at 150 v for 1,5 h. The gel was incubated with Coomassie blue stain for 30 minutes and distained for 2 hours.

### Immunizations

Adult Wistar rats (Taconic) were injected via the sub-cutaneous route with 40 μg of recombinant protein in PBS and Freund’s complete adjuvant (Sigma-Aldrich) or Alhydrogel (Statens Serum Institute) followed by two booster injections of 20 μg of protein in Freund's incomplete adjuvant or Alhydrogel at 3-week intervals [20]. Immunizations were given to groups of three animals and sera were collected and pooled 14 days after the final boosting injection.

### Animal welfare

Animal experiments complied with national and international rules for vaccination, handling, daily care and welfare. The use of Freund’s complete adjuvant has been abandoned in Denmark and immunizations with this adjuvant were performed prior to this. Animals were euthanized with Hypnorm (Vetapharma) prior to blood sampling and sacrificed with Phenobarbital (Sigma-Aldrich).

### Ethical Statement

The study was approved by the Danish Animal Experiments Inspectorate. Approval number: 2013-15-2934-00902/BES.

### Binding assays

Tritium-labeled late-stage IE (200,000 cells) and 12 μl serum were mixed in a total volume of 120 μl RPMI in triplicate wells previously coated with 2 μg/ml of Decorin (Sigma-Aldrich) and blocked with 2% bovine serum albumin (Sigma) as described [20]. After incubation for 90 min at 37°C, unbound IEs were washed away by a pipetting robot (Beckman-Coulter). The counts per minute (CPM) recording the number of adhering IE was determined by liquid scintillation counting on a Topcount NXT (Perkin-Elmer). To enable comparison of assays all data was adjusted by dividing with the mean CPM of IE incubated with pooled sera from non-immunized rats. All assays were performed twice with triplicate measurements.

### Selection of Monoclonal S2 cell lines

Drosophila S2 cells (ExpreS2ion) were transfected with the ID1-ID2a-FCR3 gene expressing pExpreS2-1 vector using ExpreS2-Insect TRx5 transfection reagent (ExpreS2ion) in Excell420 culture media (Sigma) in a 25ml T-flask. Two hours later Foetal Bovine Serum (FBS) was added to a final concentration of 10% and the cells were then incubated at 25°C. A stable polyclonal cell line was established by adding Zeocin antibiotic (Invivogen) (3000 μg/ml) to the cells 24 hours post-transfection, and maintaining the culture in 3000μg Zeocin/ml Excell420 with 10% FBS for 3 weeks. Hereafter, the established polyclonal cell line was diluted and seeded at an estimated concentration of one cell for every three wells in 96-well plates. Each well contained a final volume of 150ul Excell420, 10% FBS and 3000μg Zeocin/ml, as well as 30E3 feeder cells (untransfected S2 cells). The plates were wrapped in Parafilm, incubated at 25°C and inspected weekly to monitor clone growth. Clones from approved 96-well plates, which were clones from plates containing less than one clone per three wells, were visually confirmed to be a single clone and then picked and transferred for further evaluation in 12-well plates and tissue culture flasks by ELISA. In 12-well plates and tissue culture flasks the Zeocin concentration was reduced to 2000 μg Zeocin/ml.

### Statistical analysis

The mean binding inhibitory capacity of antibodies against H5 and S2 derived antigens was tested by Wilcoxon signed rank test (Fig 1). Statistical analysis was performed using Sigma Stat (Systat Software).

## Supporting Information

S1 Fig Purity of the recombinant proteins used for animal immunizations. Histidine tagged recombinant proteins were purified on a Ni2+-Sepharose column followed by a size exclusion column using an ÄKTA-express purification system. 15 μl of the size exclusion purifed protein fractions with higher absorbance measured in the ÄKTA-express system were analyzed in Coomassie-stained SDS gels in non-reducing conditions. In this figure, we show 3D7 NTS-ID2a protein produced in S2 cells as an example of the general purity of the proteins used in this study. F1 to F6 show the different collected fractions. F6* has the correct molecular size (116 kDa) and is the most pure fraction, so it was dialyzed to 20 mM Tris, 0.2 M NaCl, pH 8 and upconcentrated to 0,3 mg/ml before being formulated with FCA and administrated to the animals.
(TIF)
