# Supplementary figures and images for: The Influence of Sub-Unit Composition and Expression System on the Functional Antibody Response in the Development of a VAR2CSA Based Plasmodium falciparum Placental Malaria Vaccine
Source: PLoS One. 2015 Sep 1;10(9):e0135406. doi: 10.1371/journal.pone.0135406 (PMC4556615; doi:10.1371/journal.pone.0135406)

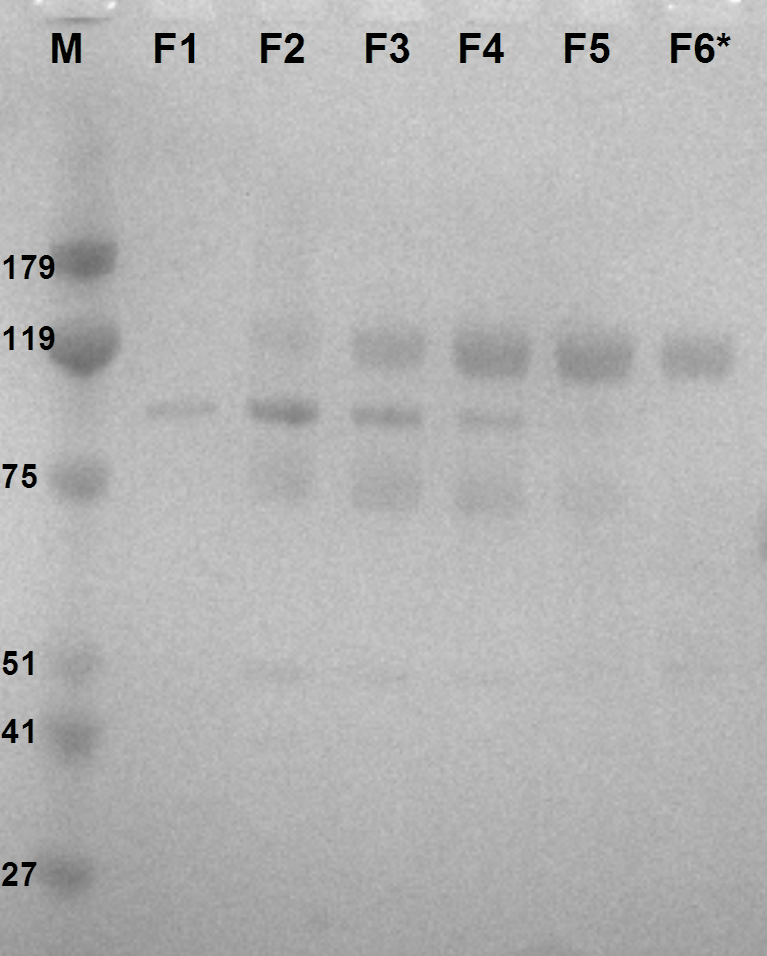

Supplement: S1 Fig — Histidine tagged recombinant proteins were purified on a Ni2+-Sepharose column followed by a size exclusion column using an ÄKTA-express purification system. 15 μl of the size exclusion purifed protein fractions with higher absorbance measured in the ÄKTA-express system were analyzed in Coomassie-stained SDS gels in non-reducing conditions. In this figure, we show 3D7 NTS-ID2a protein produced in S2 cells as an example of the general purity of the proteins used in this study. F1 to F6 show the different collected fractions. F6* has the correct molecular size (116 kDa) and is the most pure fraction, so it was dialyzed to 20 mM Tris, 0.2 M NaCl, pH 8 and upconcentrated to 0,3 mg/ml before being formulated with FCA and administrated to the animals. (TIF) [file pone.0135406.s001.TIF]
